# Welcome to *Journal of Ethnobiology and Ethnomedicine*

**DOI:** 10.1186/1746-4269-1-1

**Published:** 2005-07-29

**Authors:** Andrea Pieroni, Lisa Leimar Price, Ina Vandebroek

**Affiliations:** 1 Department of Pharmacy, School of Life Sciences, University of Bradford, Richmond Building, Richmond Road, Bradford BD7 1DP, UK; 2 SCH Group, Department of Social Sciences, Wageningen University, PO Box 8060, NL-6700 DA Wageningen, Netherlands; 3 Institute of Economic Botany, The New York Botanical Garden, Kazimiroff Blvd. & 200 Street, Bronx, New York 10458, USA

## Abstract

Ethnobiology is a multidisciplinary field of study that draws on approaches and methods from both the social and biological sciences. Ethnobiology aims at investigating culturally based biological and environmental knowledge, cultural perception and cognition of the natural world, and associated behaviours and practices. Ethnomedicine is concerned with the cultural interpretations of health, disease and illness and also addresses the health care seeking process and healing practices. Research interest and activities in the areas of ethnobiology and ethnomedicine have increased tremendously in the last decade. Since the inception of the disciplines, scientific research in ethnobiology and ethnomedicine has made important contributions to understanding traditional subsistence and medical knowledge and practice. The *Journal of Ethnobiology and Ethnomedicine *(*JEE*) invites manuscripts and reviews based on original interdisciplinary research from around the world on the inextricable relationships between human cultures and nature, on Traditional Environmental Knowledge (TEK), folk and traditional medical knowledge, as well as on the relevance of the above for Primary Health Care (PHC) policies in developing countries.

## Ethnobiology and Ethnomedicine

Ethnobiology is a multidisciplinary field of study that draws on approaches and methods from both the social and biological sciences. "Ethnobiology" has proven a rather difficult term to define since the scope of ethnobiological studies has changed considerably throughout history. One of its more recent definitions refers to the study of the reciprocal relationships between human cultures and the natural world [1]. Reciprocal relationships here refer to the human perception of the biological environment, which will ultimately influence man's behaviour, while human behaviour in turn influences – or shapes – the biological environment. This broad definition of ethnobiology encompasses ethnotaxonomy (study of the classification principles of animals, plants, soils, and ecosystems according to local peoples), ethnomedicine (study of the cultural concepts of health, disease and illness, and of the nature of local healing systems), ethnoecology (study of traditional environmental knowledge and of anthropogenic effects on the environment), ethnoagronomy (study of subsistence economies and resource management), and material culture (study of biological resources used in art and technology).

Ethnobiology aims at investigating culturally-based biological and environmental knowledge, cultural perception and cognition of the natural world, and associated behaviours and practices.

Ethnomedicine is concerned with the cultural interpretations of health, disease and illness and also addresses the health care-seeking process and healing practices.

In both ethnobiology and ethnomedicine, the documentation of the consequences of particular behaviours and practices is through cultural and biological expertise intrinsic to the fields of anthropology and biology/medicine.

## Why a new journal?

Research interest and activities in the areas of ethnobiology and ethnomedicine have increased tremendously in the last decade. The number of research publications has doubled and the three international, widely recognised scientific societies involved in this area (the Society for Economic Botany, the International Society of Ethnobiology and the International Society of Ethnopharmacology) have witnessed a continuous growth in their memberships. These three societies held their first joint scientific congress in June 2004 in the UK. .

Over the last ten years there has been a phenomenal growth of interest in indigenous, folk and local knowledge and practices among international organizations and the international development community, which include the World Health Organization (WHO), the Food and Agricultural Organization (FAO) and the World Bank. This interest is rooted in the growing understanding that indigenous knowledge and practices have a substantial value in the fields of agriculture, environmental management and biodiversity conservation, and health care. There is an increasing awareness that indigenous and local knowledge should be understood and utilized in any attempt to enhance human welfare. Importantly, the UNESCO *Convention for Safeguarding of the Intangible Cultural Heritage *that was signed at the 32^nd ^Session of UNESCO in Paris on 17 October 2003 includes the domain of knowledge and practices concerning nature and the universe. These developments convey a clear message that development paradigms are changing, recognizing that development must be considered in a manner that includes intellectual and spiritual traditions as well as indigenous and local peoples' rights to self-determination.

Since the inception of the disciplines, scientific research in ethnobiology and ethnomedicine has made important contributions to our understanding of traditional subsistence and medical knowledge and practice. More recently, the focus has increasingly been on the issues of human well-being and environmental sustainability. The boundaries between ethnobiology and ethnomedicine are increasingly merging to address complex questions. An example includes the fight against malaria and the need to understand the complex interrelationships between cultural practices, knowledge and the environment. This requires an understanding of how local practices in agriculture facilitate or counter the spread of the vector, how people understand the life cycle of the mosquito and the epidemiology of malaria, how malaria symptoms are culturally identified and interpreted, and what kind of traditional medical treatments are being used, as well as how effective these treatments are [2]. On the other hand, the use of medicinal plants for health care can no longer be seen separately from the threats posed on biodiversity since the decrease of biodiversity will ultimately affect the use of medicinal plants as health care resources.

There is a growing demand for access to integrated empirical studies in ethnobiology and ethnomedicine beyond the scope of the journals that are currently available. Much of the scientific literature in this interdisciplinary area can be categorized as being spread throughout a number of different scientific journals, where often ethnobiology and ethnomedicine are not the primary focus. While some journals do focus on ethnobiology, or on certain aspects of ethnomedicine [3], a widely available, easily accessible reference journal that specifically focuses on and integrates these two fields has been lacking. The *Journal of Ethnobiology and Ethnomedicine *will fill this void. It is our opinion that a new free-access journal in this area, sustained by a large part of the world-wide community of scholars who are working in these fields and the Editorial Board , will improve the circulation and exchange of high quality research in ethnobiology and ethnomedicine. Furthermore, we believe that the free and unhindered flow of information will not only promote a rapid increase in scientific knowledge but will also allow science to better serve the public community as a whole.

## Research areas touched by the Journal of Ethnobiology and Ethnomedicine

The *Journal of Ethnobiology and Ethnomedicine *(*JEE*) invites manuscripts and reviews based on original interdisciplinary research from around the world on the inextricable relationships between human cultures and nature, on Traditional Environmental Knowledge (TEK), folk and traditional medical knowledge, as well as on the relevance of the above for Primary Health Care (PHC) policies in developing countries.

Topics include ethnobotany, ethnozoology, ethnoecology (including ethnopedology and ethnoclimatology), ethnopharmacy (ethnopharmacognosy/folk Materia Medica), ethnomedicine, ethnoveterinary, Traditional Medicines (TMs), traditional health care in households and domestic arenas, migrant health care/urban ethnobiology, pluralistic health care in developing countries, evidence-based community health, visual ethnobiology and ethnomedicine, gender studies and ethnobiology as well as other related areas in nutritional, medical and visual anthropology. Manuscripts focussing exclusively on phytopharmacological or phytochemical investigations of traditionally used biological materials do not fit in the scope of the journal, nor do papers purely about paleobotanical, agricultural or botanical studies. The journal will be particularly keen to draw the attention of external stakeholders (public health actors; international, national and local environmental and health institutions; development agencies and NGOs) to the relevance of the traditional management of the environment in biological and cultural conservation.

Potential readers of the journal are ethnobiologists, environmentalists, ethnopharmacists, physicians, veterinarians, health care specialists, medical, cognitive, and cultural anthropologists, development workers, and policy makers.

## Peer review policy of the JEE

The *JEE *is a peer-reviewed journal. The entire reviewing process will be managed and recorded electronically. Each submission will go through a pre-evaluation process in which the Editor-in-Chief and one of the Editors (a native English speaker) will provide a cursory examination for basic English language adequacy, evaluate whether the manuscript fits the scope of the journal, and check the relevance of the manuscript to the international scientific audience of the *JEE*. This process will take one week.

Thereafter, authors of manuscripts deemed inappropriate will be informed. Authors of manuscripts with English language problems that fit within the scope of the journal will be informed and welcomed to resubmit their manuscripts after language editing. Manuscripts that do not fit broadly within the scope of the journal will be rejected. Manuscripts passing the initial pre-screening will go through the peer review process. This process should last no longer than four weeks (except in the cases of requests for major revisions).

The Editor-in-Chief will select one Deputy/Associate Editor as Managing Editor in charge of the peer review process for the manuscript. The Editor-in-Chief can also act as Managing Editor.

The Managing Editor will send the manuscript to two appropriate scholars who are internationally recognized experts in the specific field(s) featured in the manuscript, including at least one of the JEE Editorial Board Members. In the case that one of the experts, or both, would decline, the Managing Editor will send the manuscript as soon as possible to other reviewers. It is the Managing Editor's responsibility to manage the process within the given timescale.

Both reviews will be double-blinded peer reviews (neither reviewers nor authors will be able to see their names/identities).

Once peer reviews are received by the Managing Editor, the Managing Editor will decide the status of the manuscript, being either:

- acceptable as it is

- acceptable with minor revisions and corrections (concerning comprehensiveness, formatting and the quality of illustrations and tables

- acceptable only after major revisions (the paper will be reconsidered after more substantial additions and re-organization)

- to be rejected

Following resubmission with revisions drafted by the author(s), it is up to the Managing Editor to decide whether the manuscript should be returned to the original reviewers in order to obtain their final comments. This will depend on the nature and extent of the revisions. The Managing Editor will then make an editorial decision on the manuscript.

In case of divergent opinions between two reviewers where the Managing Editor is unable to make a final decision, he/she will ask for assistance from one of the Associate or Deputy Editors of the journal.

The Managing Editor will then send the peer reviews and the editorial decision to the Editor-in-Chief for final approval. The final decision communicated to authors will be sent out by the Editor-in-Chief.

If the Managing Editor's final decision diverges from the overall view of the Editor-in-Chief, the Editor-in-Chief will ask for the assistance of one of the Deputy Editors. The final decision will be taken by the Editor-in-Chief.

When the Managing Editor assigned to the manuscript is unable to meet the proposed deadlines for handling the manuscript, the Editor-in-Chief will select another Managing Editor for the manuscript or will act in some cases as (operative) Managing Editor.

## What Open Access means

The *Journal of Ethnobiology and Ethnomedicine *Open Access policy changes the way in which articles are published. First, all articles become freely and universally accessible online, so an author's work can be read by anyone at no cost. Second, the authors hold copyright for their work and grant anyone the right to reproduce and disseminate the article, provided that it is correctly cited and no errors are introduced [4]. Third, a copy of the full text of each Open Access article is permanently archived in other online repositories separate from the journal. *JEE*'s articles are archived in PubMed Central [5], the US National Library of Medicine's full-text repository of life science literature, and also in repositories at the University of Potsdam [6] in Germany, at INIST [7] in France and in e-Depot [8], the National Library of the Netherlands' digital archive of all electronic publications.

Open Access has four broad benefits for science and the general public. First, authors are assured that their work is disseminated to the widest possible audience, given that there are no barriers to access their work. This is accentuated by the authors being free to reproduce and distribute their work, for example by placing it on their institution's website. Some studies have suggested a correlation between Open Access, higher citations, and higher Impact factors [9]. Second, the information available to researchers will not be limited by their library's budget, and the widespread availability of articles will enhance literature searching [10]. Third, the results of publicly funded research will be accessible to all and not just those with access to a library with a subscription. As such, Open Access could help to increase public interest in, and support of, research. The NIH policy on public access to research calls on researchers to release to the public articles from research supported by NIH in the USA as soon as possible, and within 12 months of final publication [11]. This policy is likely to be replicated by other major funders worldwide. Fourth, a country's economy will not influence its scientists' ability to access articles because resource-poor countries (and institutions) will be able to read the same material as wealthier ones (although creating access to the internet is another matter [12]).

## Competing interests

The author(s) declare that they have no competing interests.
